# Improved respiratory motion self-gating in cardiovascular MRI

**DOI:** 10.1186/1532-429X-18-S1-P7

**Published:** 2016-01-27

**Authors:** Yu Gao, Ziwu Zhou, Fei Han, Paul J Finn, Peng Hu

**Affiliations:** 1 grid.19006.3e0000000096326718Radiology, University of California, Los Angeles, Los Angeles, CA USA; 2 grid.19006.3e0000000096326718Physics and Biology in Medicine, University of California, Los Angeles, Los Angeles, CA USA

## Background

Respiratory motion compensation is often required in cardiovascular MRI applications especially when the scan time is not suitable for breath-hold. Respiratory self-gating (RSG) is a technique that estimate the respiratory motion based on k-space signal from the imaging object and is especially advantageous in CINE-like applications where conventional diaphragm navigator frequently interrupted the image acquisition. Previously proposed RSG approaches use different algorithms, including cross-correlation (CC), center-of-mass (CM), and principle component analysis (PCA)^1,2^, to process the acquired RSG signal. However, the estimated motion in these approaches is often modulated by cardiac motion. Moreover, a quantitative evaluation on the accuracy of the estimated motion is missing due to the lack of gold-standard. In this study, we propose a template cross-correlation (TCC) algorithm that provide accurate motion estimation with little cardiac motion modulation and evaluate it, along with other algorithms, using ventilator air-way pressure signal as gold standard in pediatric patients who underwent cardiac MRI exams under general anesthesia and mechanical ventilation.

## Methods

**Figure 1:**
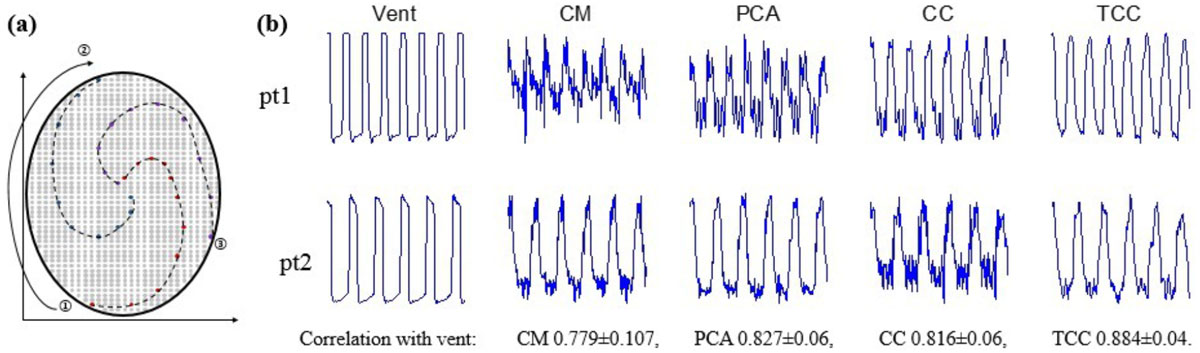
**(a): K-space sampling in ky-kz plane was ordered in a ROtating Cartesian Kspace (ROCK) manner, with each spiral arm represents one acquisition segment**. A center ky-kz (SI projection) line was inserted at the beginning of each segment for respiratory self-gating. (b): Motion extraction and correlation with ventilator results of the four algorithms on two representative patients. CM and PCA for patient 1 are highly oscillating which is undesirable without additional filter. TCC provided smoother respiratory profile and has the highest correlation with ventilator signal.

Six congenital heart disease pediatric patients under general anesthesia with mechanical ventilator support were included in this study. A high bandwidth, 3D, GRE sequence was used in this study with k-space sampling in a ROtating Cartesian Kspace (ROCK) manner^3^. A center ky-kz (SI projection) line was inserted at the beginning of each segment for respiratory self-gating (Fig. 1). Four motion extraction algorithms were compared in this study: CM, PCA, CC, and proposed TCC where instead of using a single projection as the reference for cross correlation, adjacent 5 projections are used as a template to find the profile shift. Performance of the four algorithms was evaluated in two aspects: correlation between extracted respiratory signal and the ventilator signal, and retrospective reconstruction image quality.

## Results

Fig. 1(b) shows the motion extraction results for two representative patients. CM and PCA fail to provide stable respiratory signal for patient 1. TCC is superior to the other three in terms of suppression of small oscillation, suspected as cardiac motion. Reliability of TCC is further confirmed by the fact that TCC always has the highest correlation with ventilator signal: 0.884 ± 0.04.

**Figure 2:**
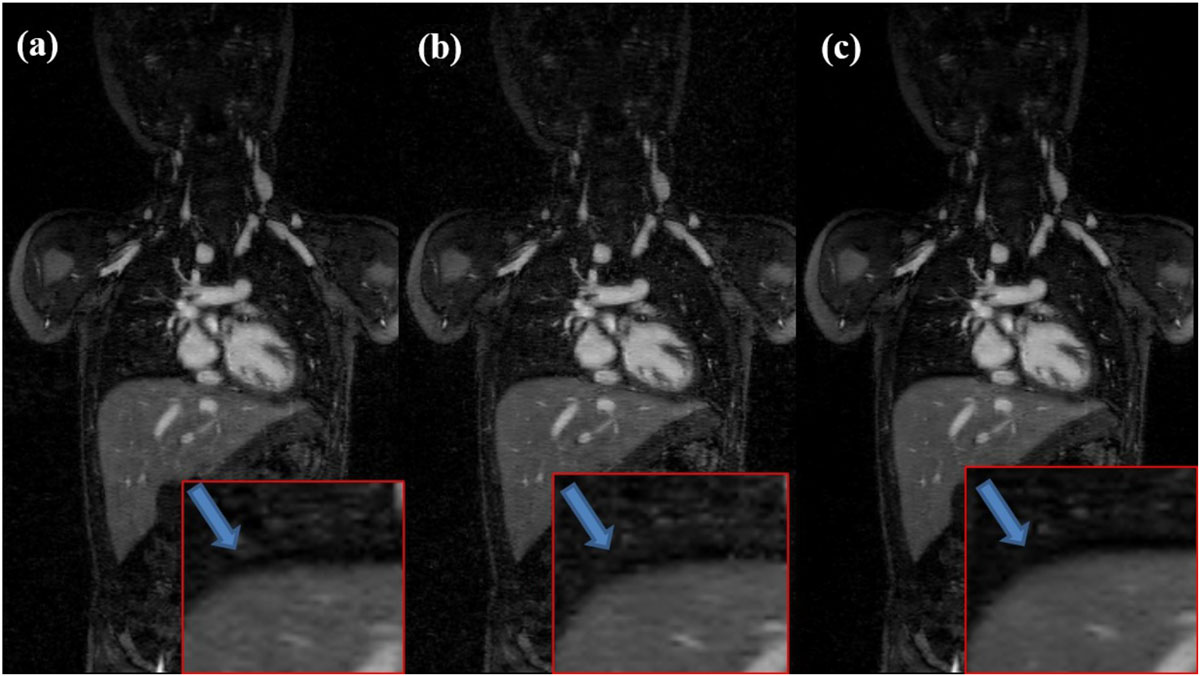
**Data acquired from an 18-month-old male with congenital heart disease**. Images were retrospectively reconstructed using L1-ESPIRiT based on ventilator signal (a), cross correlation (b) and template cross-correlation (c).

Images reconstructed with respiratory self-gating signal from TCC are comparable with, if not better than, that reconstructed from gold-standard ventilator signal. Efficacy of cardiac motion modulation elimination is visible from Fig. 2: TCC provides shaper and clean diaphragm compared with CC, which proves that TCC provides more accurate motion estimation.

## Conclusions

Incorporating temporal information in cross correlation intrinsically suppresses cardiac oscillation, and provides more reliable respiratory motion signal, which can be used to achieve high quality respiratory artifact free cardiac imaging.

